# Compliance of Health Care Workers with Hand Hygiene Practices: Independent Advantages of Overt and Covert Observers

**DOI:** 10.1371/journal.pone.0053746

**Published:** 2013-01-14

**Authors:** Sung-Ching Pan, Kuei-Lien Tien, I-Chen Hung, Yu-Jiun Lin, Wang-Huei Sheng, Ming-Jiuh Wang, Shan-Chwen Chang, Calvin M. Kunin, Yee-Chun Chen

**Affiliations:** 1 Center for Infection Control, National Taiwan University Hospital, Taipei, Taiwan; 2 Department of Internal Medicine, National Taiwan University Hospital, Taipei, Taiwan; 3 Department of Anesthesiology, National Taiwan University Hospital, Taipei, Taiwan; 4 Department of Hospital Planning, National Taiwan University Hospital, Taipei, Taiwan; 5 Department of Medicine, National Taiwan University College of Medicine, Taipei, Taiwan; 6 Departments of Internal Medicine, Ohio State University, Columbus, Ohio and University of Arizona, Tucson, Arizona, United States of America; Amphia Ziekenhuis, The Netherlands

## Abstract

**Background:**

Evaluation and feedback of hand hygiene (HH) compliance are important elements of the WHO multimodal strategy for hospital infection control. Overt observation is recommended, but it may be confounded by Hawthorne effect. Covert observation offers the opportunity to decrease observer bias. In this study we conducted a one year hospital-wide HH promotion program that included medical students (MS) as covert observers.

**Methods:**

HH compliance for the five WHO indications was determined by trained and validated observers. The overt observers consisted of eleven infection control nurses (ICNs) and two unit HH ambassadors (UAs) in each of 83 wards. The covert observers consisted of nine MS during their rotating clinical clerkships. Feedback was provided to department heads and staff each quarter.

**Results:**

Of the 23,333 HH observations 76.0% were by MS, 5.3% by ICNs and 18.7% by UAs. The annual compliance rates were MS 44.1%, ICNs 74.4% and UAs 94.1%; P<0.001. The MS found significantly lower annual compliance rates for 4/5 HH indications compared to ICNs and UAs; P<0.05. The ICNs reported significantly improvement from the first to the fourth quarter; P<0.001. This was associated with feedback from the MS of very poor compliance by nurses during the first quarter.

**Conclusions:**

Based on these findings we recommend a two-pronged approach to HH programs. The role of ICNs and UAs is to educate, serve as role models, establish, sustain good HH practices and provide direct feedback. The role of the covert observers is to measure compliance and provide independent feedback.

## Introduction

The World Health Organization (WHO) launched the global hand hygiene (HH) program in 2004 to reduce healthcare-associated infections (HAIs) and improve patient safety. Evaluation and feedback of HH performance are important elements of this program. The WHO Guidelines on Hand Hygiene in Health Care and the Centers for Disease Control and Prevention, USA recommend direct observation of compliance and measuring the consumption of HH products [1]–[3]. Direct observation helps to pinpoint areas of strength or weaknesses in HH behavior, identify the number of HH opportunities, their indications, assess technique and provide feedback to healthcare workers (HCWs) [4], [5]. HH performance is usually improved when the HCWs know that they are under observation. These changes in behavior are often attributed to the well-known “Hawthorne effect” [6].

In a recent editorial commentary Daniels emphasized the need to reconsider our approach to monitoring hand washing [7]. He pointed out that HCW compliance rates with HH practices remain unacceptably low, and that there are multiple problems with the current gold standard of direct observation. These include “ investment in human capital all but ensures that undersampling will occur”, direct observations are limited to work shifts, and “secret observers” who are not part of the health care team will eventually be detected. He was intrigued by the report of Hornbeck et?al. [8] in which the authors described the use of a mote-based sensor to record contacts among HCWs and their important observation that HCW contact patterns dramatically affects disease diffusion.

We also have been concerned that observation bias, cueing and peer pressure overestimates the rates of compliance. The current study was designed to evaluate the potential problems of observation bias by comparing the rates of HH compliance by covert overt as well as overt observers. It was conducted as part of a one year hospital-wide HH promotion program at a large teaching medical center in Taipei, Taiwan.

We invited medical students (MS) to serve as covert observers during their clinical clerkships. We reasoned that since MS are an integral part of the health care team they would have multiple opportunities to unobtrusively observe HH practices by HCWs during daily rounds. Infection control nurses (ICNs) and unit HH ambassadors (UAs) served as overt observers. All three groups received the same training and used the same data collection methods.

## Materials and Methods

### Hospital Setting and Hand Hygiene Program

National Taiwan University Hospital (NTUH) is a 2200-bed major teaching hospital in Taipei, Taiwan that provides both primary and tertiary medical care. Approximately one third of the hospital’s house staff are replaced each year. The distribution and time trends of HAIs and infection control programs during 1981 to 2007 have been described previously [9]. An alcohol-based hand rub was introduced in 2004 and a hospital-wide hand hygiene (HH) program was implemented and promoted annually [10], [11]. The program was reviewed, revised and promoted annually in accordance with plan-do-check-act cycle based on the performance in the preceding year. The WHO multimodal hand hygiene improvement strategy was adapted in 2009. A detailed description of the action plan and hand washing campaigns is provided in appendix S1. The five WHO indications for HH are listed at the bottom of Table 1 [4].

**Table 1:** The distribution of opportunities for three types of observers to assess hand hygiene program during a one-year study conducted during 2010 and 2011 by the Center of Excellence for Hand Hygiene (HH) at the National Taiwan University Hospital.

	Number (%) of hand hygiene opportunities observed			
Parameter	Total	Medical students	Infection control nurses	Unit HH ambassadors
Total opportunities observed*	23333	17742	1228	4363
Professional category†				
Doctors	14656 (62.8)	12788 (72.1)	255 (20.8)	1613 (37.0)
Nurses	5481 (23.5)	2029 (11.4)	869 (70.8)	2583 (59.2)
Others	3196 (13.7)	2925 (16.5)	104 (8.5)	167 (3.8)
Department†				
Medical	7452 (31.9)	5592 (31.5)	410 (33.4)	1450 (33.2)
Surgical	3409 (14.6)	2286 (12.9)	281 (22.9)	842 (19.3)
Pediatrics	152 (0.7)	9 (0.1)	29 (2.4)	114 (2.6)
Gynecological	2268 (9.7)	1571 (8.9)	142 (11.6)	555 (12.7)
Others	10052 (43.1)	8284 (46.7)	366 (29.8)	1402 (32.1)
**Hand hygiene indications** †				
1. Before touching a patient	8996 (38.6)	7418 (41.8)	229 (18.6)	1349 (30.9)
2. Before clean/aseptic procedure	1598 (6.8)	462 (2.6)	172 (14.0)	964 (22.1)
3. After body fluid exposure risk	1278 (5.5)	423 (2.4)	221 (18.0)	634 (14.5)
4. After touching a patient	9802 (42.0)	7612 (42.9)	526 (42.8)	1664 (38.1)
5. After touching patient surroundings	3759 (16.1)	2983 (16.8)	193 (15.7)	583 (13.4)

* The differences among the observers in frequency of opportunities to witness HH performances were significant; P<0.001.

† The differences among the observers in proportion of observations according to professional categories, department or HH indications were significant; P<0.001.

### Overt and Covert Observers

The overt observers consisted of eleven experienced ICNs and two UAs on each ward. The UAs consisted of a physician and a nurse who were worked on the unit under study. They were responsible for education, communication, audit and immediate feedback of HH performance by the HCWs on their ward. The ICNs and UAs wore uniforms and representative badges on their chests. Some stated their purpose before making their observations. They observed HH practices of physicians, nurses and other HCWs on 83 in-patient units: 24 medical, 18 surgical, 11 pediatric, 4 gynecological wards, and 26 other wards including orthopedics and urology.

The covert observers consisted of nine 5th to 7th year volunteer MS who rotated on the medical, surgical, gynecological and pediatric services and made ward rounds with the assigned medical team. The staff of the units under study were not aware of their identify as covert observers.

### Training and Validation of Observers

The observers were required to take the same basic course as other HCWs in HH practices. In addition they participated in a 2-hour workshop, conducted by the same trainer, using WHO standard training materials. A WHO training film included in the WHO Implementation Toolkit [12] was used to provide case scenarios of the five moments for HH. A standardized hand hygiene observation test, modified from “Hand-hygiene Observation Tool” test developed in UK, was then used to evaluate the participants [13]. The performance of students, ICN, and UAs were 100±0, 70.4±9.7, and 72.0±10.4, respectively (total score 100). The answers to each question were provided following the tests. The trainer facilitated discussions by the participants to clarify the concepts provided in slide presentation that accompanied the film.

### Evaluation and Feedback

A standard data form was used to record compliance by HCWs with the five WHO HH indications. The data included hospital unit, professional category (physicians, nurses and other HCWs) and clinical department. The HCWs were not identified to protect their privacy. Unit and department-specific HH compliance rates were evaluated each month by the investigators. The data were provided to the chief physician and head nurse of the unit and department directors each quarter. In addition to periodic feedback, we re-educated the HH UAs in March 2011 to emphasize weaknesses identified during their audits.

### Hand Hygiene Product

The hand hygiene products included an alcohol based hand rub (Clean Anti-bacterial Hand Sanitizer, Panion & BF Biotech Inc., Taiwan), chlorhexidine (Hibiscrub, Molnlycke Healthcare, UK), and liquid soap (Luis Pearly Liquid Soap, Sa-Toun, Taiwan). To improve the accessibility of the hand hygiene products, the location of the alcohol-based hand rub was increased from the first quarter from one per multi-bed room to at least one for every 2 beds.

The consumption of hand hygiene products was monitored by the amount sent to each ward by the Department of General Affairs.

### Data Analysis and Statistics

A single individual entered all the data from hard copy onto a standardized spreadsheet. Compliance was defined as the ratio of the number of performed actions to the number of HH opportunities [4]. The Chi square test was used to test the difference in compliance rates by type of observers, quarter, professional category, department and the HH indication.

### Ethics Statement

This study was approved by the Institutional Ethics Review Board of the National Taiwan University Hospital (No. NTUH- 201109015RC) and the requirement for informed consent from each patient was waived due to the project was part of the hospital-wide quality improvement program and an observational study.

## Results

The distribution of the opportunities to observe HH performance, during the study year, by type of observer, HCW category and WHO indication are shown in Table 1. The MS made 76.0% of the 23,333 observations, followed by the UAs (18.7%) and ICNs (5.3%). The differences among the observers in frequency of opportunities to witness HH performances were highly significant; P<0.001. The most frequent indications for HH were before (38.6%) and after touching a patient (42.0%). These were followed by after touching the patients surroundings (16.1%), before a clean aseptic procedure (6.8%) and after exposure to body fluids (5.5%). The MS were less likely than ICNs and UAs to witness aseptic procedures and body fluid exposures, both *p<0.001*.

**Figure 1:**
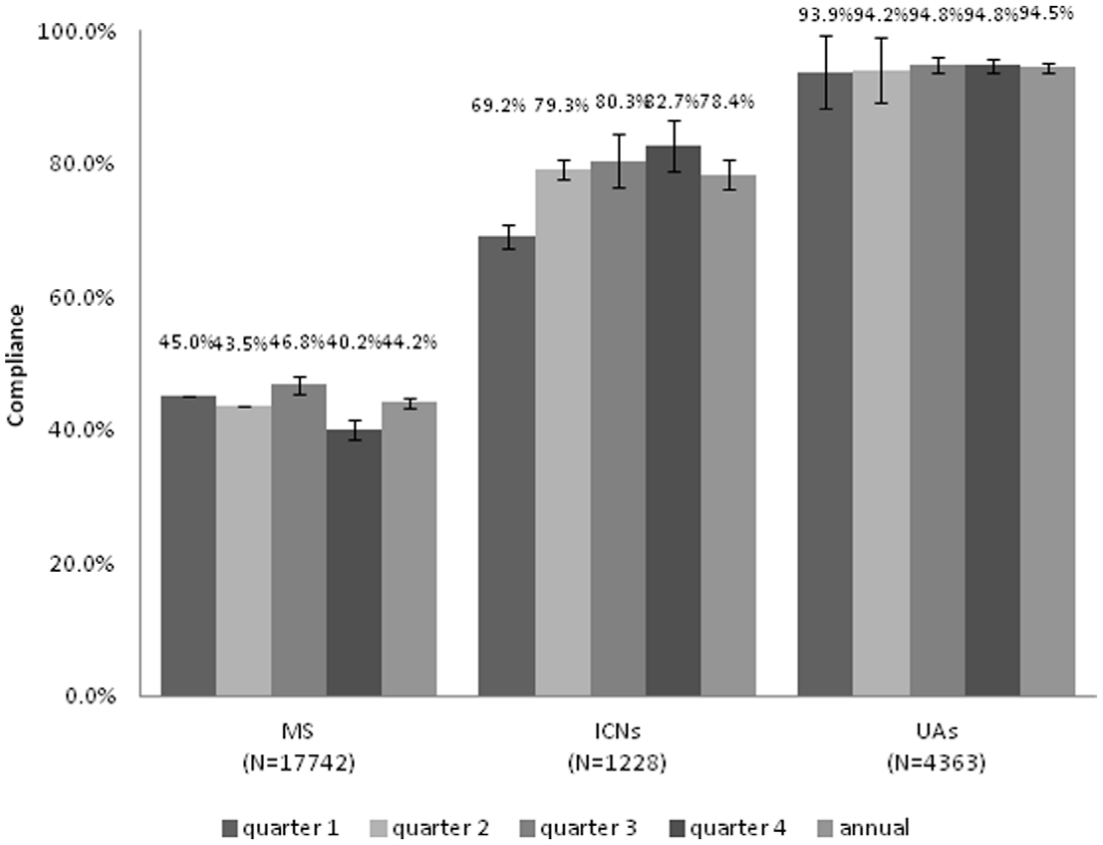
Hand hygiene compliance rates by type of observers and quarter. The compliance rates observed by medical students (MS) were significantly lower than those by infection control nurses (ICNs) and unit HH ambassadors (UAs) in each quarter (all the *P* value <0.001). The numbers in parenthesis represented hand hygiene opportunities observed. T-bar represented one standard deviation.

The overall HH compliance rates by type of observer for each quarter and the study year are shown in Figure 1. The quarterly and annual compliance rates observed by the MS were significantly lower than those of the ICNs and UAs (44.1% versus 74.4% and 94.1%, respectively; P<0.001). The MS found significantly lower annual compliance rates for 4/5 HH indications compared to ICNs and UAs; P<0.05. The ICNs noted significant improvement in quarterly compliance rates from the first to last quarter, p<0.001. This was associated with feedback from the MS of poor HH compliance by the nurses during the first quarter (see **Feedback** **and improvement**).

**Figure 2:**
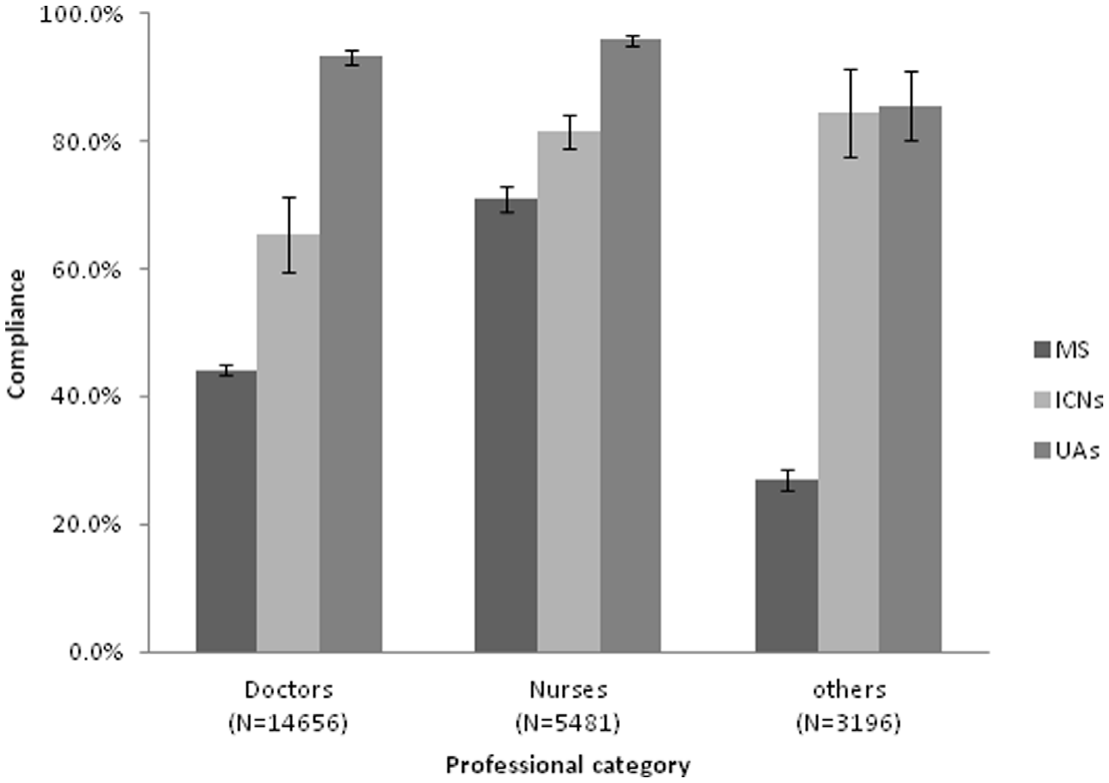
Hand hygiene compliance rates according to professional category of the healthcare workers and type of observers. Compliance observed by medical students (MS) was significantly lower as than by infection control nurses (ICNs) and unit HH ambassadors (UAs) (all P<0.001). The numbers in parenthesis are the hand hygiene opportunities. T-bar represents one standard deviation.

The HH compliance rates, during the study year, according to professional category and type of observer are shown in Figure 2. The MS reported significantly lower compliance rates than ICNs and UAs for all professional categories; P<0.001. Both the students and ICNs reported lower compliance rates for doctors than nurses; both P<0.001.

### Feedback and Improvement

**Figure 3:**
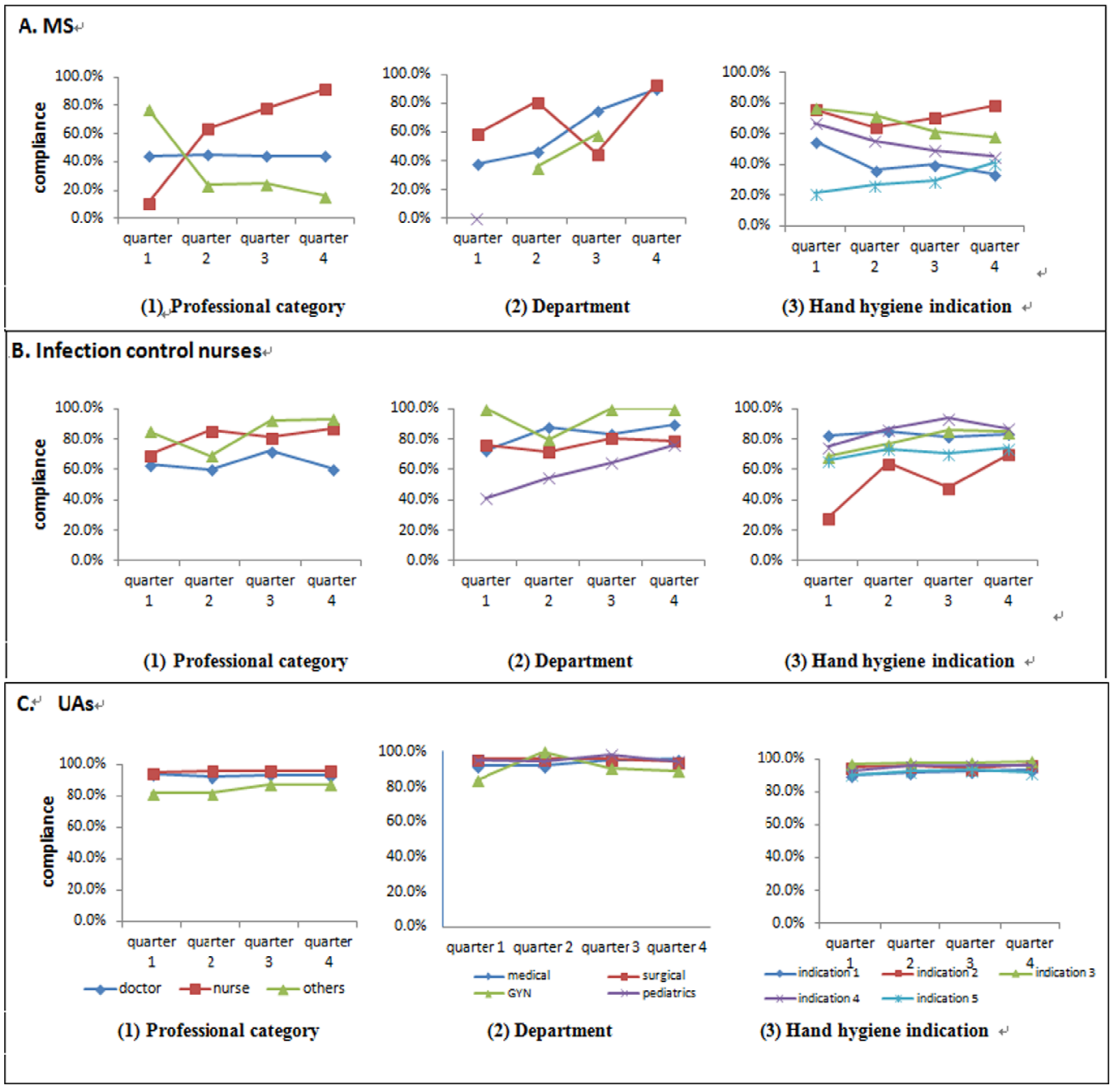
Hand hygiene compliance rate by type of observers (A. medical student (MS), B. Infection control nurses, C. unit HH ambassadors (UAs)), professional category, department, indication of hand hygiene, and period.

Feedback of the HH practices was provided to staff by ICNs and UAs each quarter with special emphasis on HH for clean procedures. The quarterly HH compliance rates, by type of observer, according to professional category, department and HH indication are shown in Figure 3. The MS reported significantly improved compliance by the nurses; P<0.001 (see above), but poorer compliance by other HCW; P<0.001. The ICNs reported significant improvement by the pediatric department; P = 0.004 and hand indication 2, P<0.001. The UAs consistently reported compliance rates of 80 to 100% for all quarters.

### Indirect Observations

The average consumption of hand hygiene products per 1000 patient-days is shown in Figure S1. These consisted of 34.9 L of alcohol-based hand rub, 13.6 L of disinfectant, and 10.3 L of liquid soap. Consumption for any of these products did not significantly change during the study period (P = 0.72, 0.24, and 0.55, respectively).

## Discussion

The key findings of this study were the ability of well-trained, volunteer MS to covertly observe three quarters of the 23,333 opportunities for HH compliance and to detect significantly lower rates of compliance than full time overt ICNs and UAs over a one-year period at a major medical center in Taiwan. In addition, the MS finding of poor compliance by the nursing staff, during the first quarter, led to feedback that significantly improved their compliance in subsequent quarters. The MS also found that the quarterly HH compliance rates did not improve despite intense educational efforts during the study year. The ability of MS to serve as covert observers to assess simple HH procedures was confirmed during their training and verified in the first quarter to be concordant with experienced ICNs. The ICNs appear to have overestimated compliance by 30.3% and UAs by 50.0% when compared to MS. These large differences between covert and overt observers question the ability of ICNs and UAs to adequately assess HH compliance.

The MS noted improved HH compliance by nurses, but not physicians despite repeated feedback during the study period. This might be explained by the cultural characteristics of busy physicians and surgeons, decreased time on duty of house staff, and changes in residency training programs.

The characteristics and pros and cons of covert and overt HH observers are summarized in Table 2. The advantages of using MS as covert observers during their clinical clerkships are: far more opportunities to witness HH practices by HCWs on day, night and weekend shifts, avoiding selection bias and cues that might lead to the Hawthorne effect, while maintaining patient privacy. It is hoped that this experience will make them more critical observers and foster the development of a cadre of physicians concerned with hospital infection control.

**Table 2:** Comparison of the characteristics of three types of observers and the pros and cons their ability to assess the efficacy of the hand hygiene program during a one-year study conducted during 2010 and 2011 by the Center of Excellence for Hand Hygiene at the National Taiwan University Hospital.

Type of observer	MS (Covert)	Infection control nurses (Overt)	UAs (Overt)
Professional level	• 5^th^ grade of 7-year MS	• Full-time infection control nurses who were not part of the unit under study	• One doctor and one nurse with patient care responsibilities in the unit under study.
	• Volunteers	• Full time employees	• Assigned by department heads and head nurses.
Observation method	• The unit staff was not aware of theiridentity	• The unit staff was aware of their identity	• The unit staff was aware of their identity
	• Observation made on rounds	• Overt observation was made during ward visits for active surveillance of healthcare-associated infections^10, 11^	• Overt observation on a random, unannounced day within a designated month
	• More focus on physicians because ofclerkship activities		• Observation made during their daily work
Number of opportunities for observation of hand hygiene	• At least 30 per week per student.	• At least 2 per ward per month duringroutine ward visits. Each nurse wasin charge of 6–8 units.	• At least 2 per month per ambassador
	• Observations made as part of the medical team on days, nights and weekends.		
Pros	• Avoid the Hawthorne effect	• Experienced professionals	• Bottom-up involvement of staff in each unit to promote hand hygiene and institution safety
	• No conflict of interest	• No conflict of interest	• Observation at any time, including during day and night-shifts
	• Educational benefit from early awareness of the importance of hand hygiene	• Immediate and direct feedback (optional)	• Immediate and direct feedback to HCWs
	• Potential advocates of HH and infection control	• Maintained HCWs privacy	• Increased hand hygiene opportunities during each observation period
	• Feedback was provided by the investigators		• Maintained HCWs privacy
	• Maintained HCWs privacy		
Cons	• No immediate or direct feedback,• Not familiar with invasive procedures	• Potential Hawthorne effect	• Potential Hawthorne effect
		• Observations made only during weekdays; not at night	• Potential to overestimate HH compliance rate
		• Potential to overestimate HH compliance	• Conflict of interest with doctors and nurses on the unit

MS: medical students; UA: unit HH ambassador.

Several investigators have reported significant improvement in infection control programs when front line ward HCWs were recruited as participants [14]–[16]. The advantages of using ICNs and UAs are their ability to promote the HH program, educate and stimulate compliance with good HH practices and provide immediate feedback [17]. In this respect the so-called Hawthorne effect should enhance the opportunity to reinforce good HH practices. The disadvantages of using ICNs to assess compliance are: fewer opportunities to witness HH by HCWs, short periods of observation and ready identification by HCWs. The disadvantage of using UAs to assess compliance is their apparent inability or unwillingness to detect poor compliance. This is most likely accounted for by peer pressure and the need to attend to more compelling duties and responsibilities.

The first hospital-wide study was conducted in 1994 by Pittet et?al. at a teaching hospital in Switzerland [16]. Infection control nurses conducted 2,834 20-minute observations on 48 wards distributed randomly during the day and night over 14 days. The average compliance was 48%. They found that noncompliance was higher among physicians and other HCWs than among nurses and was lowest on weekends. Noncompliance was higher in intensive care than internal medicine units and during procedures that carried a high risk for contamination. Our findings, using MS as covert observers, were virtually identical to those of Pittet et?al. The annual compliance rates were 44.1% and physicians were found to be less compliant than nurses.

We are aware of only two studies that used staff volunteers or research nurses as covert observers. Both were conducted in the ICU setting [3], [6]. ICUs are structured quite differently from regular hospital rooms. They tend to be relatively open areas or small self-contained units. ICUs provide numerous opportunities for patient contact with multiple personnel of different levels of training and experience as well as exposure to contaminated instruments and fluids [8]. The ICU architecture allows research nurses to make observations at a distance without revealing their identities [6]. In contrast, most hospital wards are structured as separate rooms containing one or more patients. It is much more difficult for observers to explain their presence. MS need not identify themselves as covert observers because they are considered as part of the medical team at the point of care. MS have previously been shown to be effective overt observers in HH programs [18]. They reduced labor costs, sustained surveillance and provided immediate feedback, but their role was similar to those of ICNs.

We believe that use of MS as covert observers, as reported in the current study, addresses several of the criticisms of overt observers in Daniels’ editorial commentary [7] and confirms the work of Pittet et?al [16]. The advantages of using MS as covert observers are: First, the investment in human capital was greatly reduced. Second, the MS were able to make a far greater number of observations than ICNs, including all work shifts and days of the week. Third, the MS were able to provide feedback to the infection control team about instances of poor compliance that was missed by the ICNs. Electronic monitoring systems add to our understanding of the consequences of exposing patients to peripatetic personnel within the complex hospital environment [8]. It remains to be seen whether electronic systems can be adapted to monitor effective hand washing practices in hospitals. Even if effective monitoring devices can be developed considerable effort would still be needed to assure compliance. In the meantime, we believe that the combination of overt and covert observers combined with effective feedback offers the most effective approach to improve hand washing practices.

This study has several strengths and limitations. The strengths include: it was conducted at a major teaching hospital experienced in promoting good HH practices; it included a very large number observations; confounding by seasonal variations was minimized by including all quarters during the study year; the MS observations were in accord with no significant change in use of HH hygiene products during the study year; and we were able to compare covert and overt observers, while maintaining HCW privacy. The limitations include: the observers were not required to record a fixed proportion of observations according to professional categories, HH indications or department. This might have resulted in selection bias in comparing quarters and among observers. To deal with this issue we used stratification analysis to show that the compliance rates observed by students were still lower than those of the overt observers, irrespective of professional category, department or HH indication. We were unable to directly compare the covert and overt observers on individual wards because of the small number of observations made by ICNs and UAs on each unit.

Future strategies include earlier incorporation of hand hygiene training in the curriculum of health care professionals and more vigorous enforcement.

In conclusion, education, observation of HH performance and feedback are key elements of an HH program. We recommend a two-pronged approach to HH programs. The role of ICNs and UAs is to educate, serve as role models, establish and sustain good HH practices and to provide direct feedback. The “Hawthorne effect” probably strengthens these efforts. The role of the covert observers is to establish efficacy independent of observer bias and the “Hawthorne effect”.

## Supporting Information

Figure S1 The monthly consumption of hand hygiene products during 2010 and 2011 at the National Taiwan University Hospital.
(TIF)

Appendix S1 The Action Plan Used as Road Map to Implement Hand Hygiene Campaigns at the National Taiwan University Hospital.
(DOCX)
