# Supplementary figures and images for: Compliance of Health Care Workers with Hand Hygiene Practices: Independent Advantages of Overt and Covert Observers
Source: PLoS One. 2013 Jan 14;8(1):e53746. doi: 10.1371/journal.pone.0053746 (PMC3544847; doi:10.1371/journal.pone.0053746)

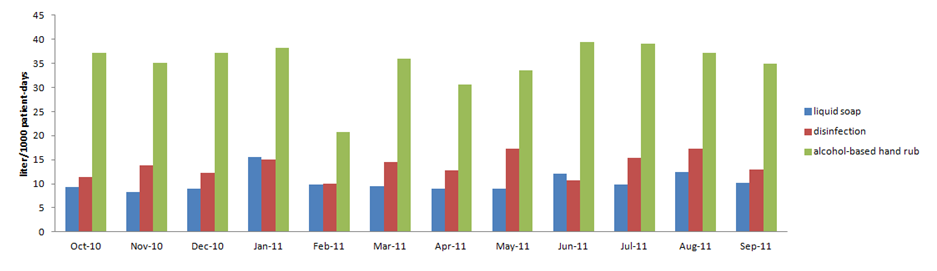

Supplement: Figure S1 — The monthly consumption of hand hygiene products during 2010 and 2011 at the National Taiwan University Hospital. (TIF) [file pone.0053746.s001.tif]
