# Supplementary material for: Compliance of Health Care Workers with Hand Hygiene Practices: Independent Advantages of Overt and Covert Observers
Source: PLoS One. 2013 Jan 14;8(1):e53746. doi: 10.1371/journal.pone.0053746 (PMC3544847; doi:10.1371/journal.pone.0053746)
Supplement: Appendix S1 — The Action Plan Used as Road Map to Implement Hand Hygiene Campaigns at the National Taiwan University Hospital. (DOCX) [file pone.0053746.s002.docx]

**Appendix S1**

**The Action Plan Used as Road Map to Implement Hand Hygiene Campaigns at the National Taiwan University Hospital**

To execute the project, sponsored by Taiwan Centers for Disease Control, we established a multidiscipline working group supervised by the vice-superintendent in October 2009. Members included personnel from the Center for Infection Control, Departments of Hospital Planning, Quality Control, Nursing, Medical Education, Public Affairs, Social Service, and Information Technology. Through sharing, coordination and accountability in program activities, more healthcare workers became familiar with the importance and impact of hand hygiene on patient safety.

**Systemic change**

A hospital-wide survey of hand hygiene facilities focused on alcohol-based hand rub dispensers and reminders was conducted in March 2010. The Infection Control Committee approved the updated “Standards for Hand Hygiene Facilities” in November 2010. The standards emphasized the accessibility of alcohol-based hand rub at the point of care, including resuscitation and work trolleys, treatment carts, and carry-on or a pocket alcohol-based hand rub that was only available on psychiatric wards. A hospital-wide survey of hand hygiene facilities was conducted every three months thereafter coupled with improvement of the facilities.

**Policies, principles and procedures**

In 2010, we incorporated hand hygiene opportunity into the updated “Infection Control Guidelines for Invasive Procedures” performed outside operating rooms. In 2011, The Infection Control Committee approved the updated “Guidelines for Hand Hygiene implementation” based on the 2009 version of WHO hand hygiene guideline. This guideline emphasizes the “My Five Moments for Hand Hygiene” approach. The first draft was published in 1996 and a recent 9^th^ revision was approved in 2009. In addition, we initiated several taskforces to conduct a review and revision of workflows and procedures for several important clinical skills. The process included literature review and discussion between infection control personnel and primary care nurses and physicians and is still ongoing.

**Education**

The education of hand hygiene program for staff and other healthcare workers were based on the “Guidelines for Hand Hygiene Implementation.” We developed an education program by level and by purpose for audients. We provided a 2-hour workshop for “Hand Hygiene" UAs (representatives from each unit) in a train-the-trainee manner. We also provided a 2-hour workshop for “mystical guests” (MS volunteer) which included a knowledge-based basic course and the skill to audit bedside hand hygiene practice.

We utilized tools in the “Guide to the Implementation of the WHO Multi-Modal Hand Hygiene Improvement Strategy” particularly the Slides for the Hand Hygiene Coordinator, Slides for Education Sessions for Trainers, Observers and Health-care workers, Hand Hygiene Training Films and Slides accompanied the training films We developed a Hand Hygiene Technical Reference Manual, Observation Forms, Your Five Moments for Hand Hygiene Poster, Hand Hygiene Technical Reference Manual and Observation Tools consisting of an observation and a compliance calculation form. We conducted ward infrastructure, soap/hand rub consumption, and perception surveys for healthcare workers, and senior managers. We wrote a Hand Hygiene knowledge questionnaire for healthcare workers, and Hand Hygiene: When and How Leaflet.

We established a one-hour lecture that was also available as an on-line e-learning system in 2004. E-learning programs include post-class evaluation. In 2010 we updated the focus of the lecture on the importance of hand hygiene (lesson from the history and current international trend), the “My 5 Moments for Hand Hygiene” approach and the correct procedures for hand washing and hand rubbing. Three lecture sessions were offered in 2010, focused on healthcare workers on the floor, administrators and supporting personnel, and the public (outpatients, family and visitors), respectively.

We established skill-based education and accreditation in May 2010 following World Hand Hygiene Day special activity. The process was conducted by “Hand Hygiene UAs” and infection control nurses in each hospital service to verify the knowledge of 5 moments for hand hygiene and focus on correct procedures of hand washing or hand rubbing. An “accreditation document” was given to each healthcare workers who passed the validation program.

World Hand Hygiene Day activities were open to the public on May 5, 2010 and 2011. More than 500 visitors attended each of these events. Our medical students introduced a 7-step hand hygiene technique using a Ku-Fung based slogan. All participants practiced hand hygiene technique and a UV detector identified the weak points in HH. Please visit our web for the detail (http://www.ntuh.gov.tw/ifc/hhc/default.aspx).
